# Diaqua-2κ^2^ *O*-bis­(μ-1-oxido-2-naphtho­ato)-1:2κ^3^ *O* ^1^,*O* ^2^:*O* ^2′^;2:3κ^3^ *O* ^2^:*O* ^1^,*O* ^2′^-bis­(1-oxido-2-naphthoato)-1κ^1^ *O* ^2^,*O* ^2^;3κ^2^ *O* ^1^,*O* ^2^-hexa­pyridine-1κ^2^ *N*,2κ^2^ *N*,3κ^2^ *N*-trimanganese(II/III) pyridine disolvate dihydrate

**DOI:** 10.1107/S1600536808038439

**Published:** 2008-11-26

**Authors:** Hua Yang, Yuting Chen, Dacheng Li, Daqi Wang

**Affiliations:** a School of Chemistry and Chemical Engineering, Liaocheng University, Liaocheng 252059, People’s Republic of China; b Department of Chemistry, Dezhou University, Dezhou 253023, People’s Republic of China

## Abstract

The title complex, [Mn_3_(C_11_H_6_O_3_)_4_(C_5_H_5_N)_6_(H_2_O)_2_]·2H_2_O·2C_5_H_5_N, is a trinuclear mixed oxidation state complex of 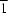 symmetry. The three Mn atoms are six-coordinated in the shape of distorted octa­hedra, each coordinated with an O_4_N_2_ set of donor atoms, where the ligands exhibit mono- and bidentate modes. However, the coordination of the Mn^II^ ion located on the inversion centre involves water mol­ecules at two coordination sites, whereas that of the two symmetry-related Mn^III^ ions involves an O_4_N_2_ set of donor atoms orginating from the organic ligands. Intramolecular C—H⋯π interactions between neighbouring pyridine ligands stabilize this arrangement. A two-dimensional network parallel to (001) is formed by inter­molecular O—H⋯O hydrogen bonds.

## Related literature

For the crystal synthesis of metal complexes with hydroxy­naphthoates, see: Schmidt *et al.* (2005 ▶); Ohki *et al.* (1987 ▶). 
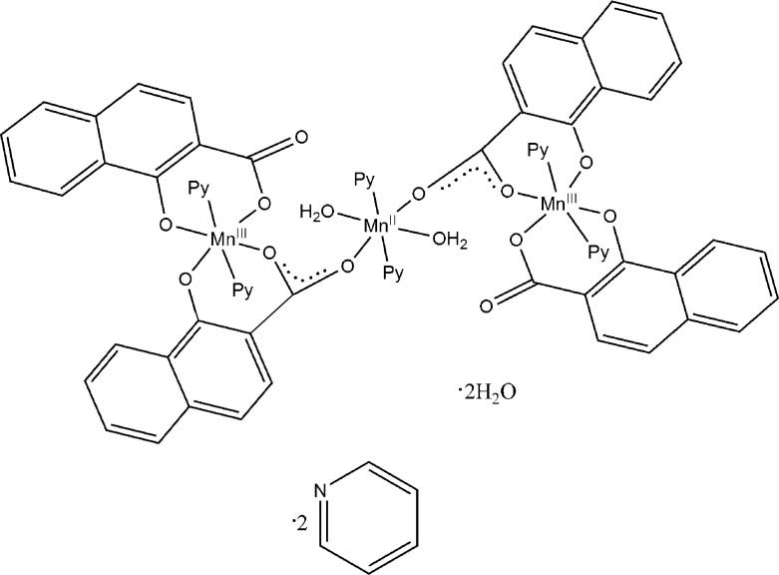


## Experimental

### 

#### Crystal data

[Mn_3_(C_11_H_6_O_3_)_4_(C_5_H_5_N)_6_(H_2_O)_2_]·2H_2_O·2C_5_H_5_N
*M* *_r_* = 1614.32
Triclinic, 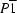

*a* = 9.962 (3) Å
*b* = 10.170 (3) Å
*c* = 19.812 (5) Å
α = 77.624 (3)°
β = 89.053 (4)°
γ = 85.370 (4)°
*V* = 1954.2 (10) Å^3^
*Z* = 1
Mo *K*α radiation
μ = 0.55 mm^−1^
*T* = 298 (2) K
0.45 × 0.44 × 0.16 mm

#### Data collection

Bruker SMART CCD area-detector diffractometer
Absorption correction: multi-scan (*SADABS*; Sheldrick, 1996 ▶) *T*_min_ = 0.790, *T*_max_ = 0.917
10152 measured reflections
6766 independent reflections
3917 reflections with *I* > 2σ(*I*)
*R*_int_ = 0.028

#### Refinement

*R*[*F* ^2^ > 2σ(*F* ^2^)] = 0.048
*wR*(*F* ^2^) = 0.154
*S* = 1.00
6766 reflections
502 parameters
744 restraints
H-atom parameters constrained
Δρ_max_ = 0.41 e Å^−3^
Δρ_min_ = −0.26 e Å^−3^

### 

Data collection: *SMART* (Bruker, 2001 ▶); cell refinement: *SAINT* (Bruker, 2001 ▶); data reduction: *SAINT*; program(s) used to solve structure: *SHELXS97* (Sheldrick, 2008 ▶); program(s) used to refine structure: *SHELXL97* (Sheldrick, 2008 ▶); molecular graphics: *SHELXTL* (Sheldrick, 2008 ▶); software used to prepare material for publication: *SHELXTL*.

## Supplementary Material

Crystal structure: contains datablocks I, global. DOI: 10.1107/S1600536808038439/kp2187sup1.cif

Structure factors: contains datablocks I. DOI: 10.1107/S1600536808038439/kp2187Isup2.hkl

Additional supplementary materials:  crystallographic information; 3D view; checkCIF report

## Figures and Tables

**Table 1:** Selected bond lengths (Å)

Mn1—O2	2.180 (2)
Mn1—O7	2.198 (2)
Mn1—N1	2.268 (3)
Mn2—O6	1.854 (2)
Mn2—O3	1.874 (2)
Mn2—O1	1.891 (2)
Mn2—O4	1.909 (2)
Mn2—N3	2.321 (4)
Mn2—N2	2.349 (4)

**Table 2:** Hydrogen-bond geometry (Å, °)

*D*—H⋯*A*	*D*—H	H⋯*A*	*D*⋯*A*	*D*—H⋯*A*
O7—H7*A*⋯O8^i^	0.85	2.03	2.799	150
O8—H8*A*⋯O5^ii^	0.85	2.04	2.889	178
O8—H8*B*⋯O5^iii^	0.85	2.13	2.981	178
C31—H31⋯*Cg*^iv^	0.93	3.22	3.847	127

Symmetry codes: (i) 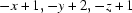; (ii) 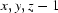; (iii) 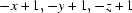; (iv) 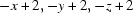. *Cg* is the centroid of the N1/C23–C27 ring.

**Fig. 1.**
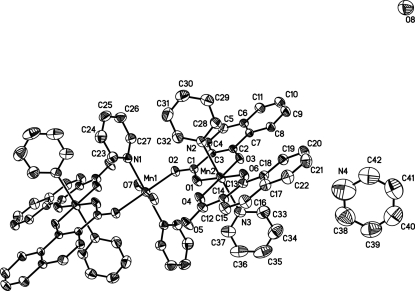
Molecular structure of the complex showing the atomic numbering and the 30% probability displacement ellipsoids. Unlabelled atoms are related to the labelled ones by symmetry operation (-x, -y, -z). C-bound H atoms have been omitted for clarity.

**Fig. 2.**
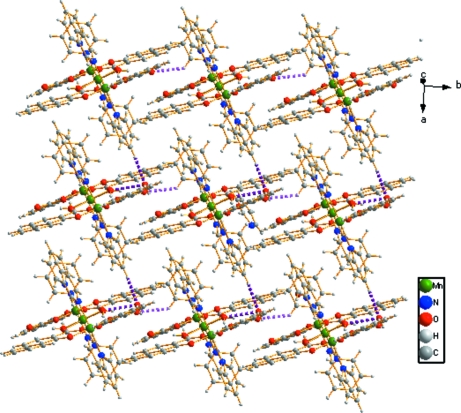
Crystal packing of (I).

## supplementary crystallographic information

### Comment

Multi-nuclear coordinated polymer attracted more attention in coordination chemistry. But transition metal complexes with hydroxynaphthoates as ligand have rarely been synthesized or crystallized except for some lanthanoids (Ohki *et al.*, 1987) and manganese complexes (Schmidt *et al.*, 2005). In this paper the synthesis of the title complex [Mn_3_(C_11_H_6_O_3_)_4_(C_5_H_5_N)_6_(H_2_O)_2_] (H_2_O)_2_(C_5_H_5_N)_2_was reported. Black-brown crystals suitable for X-ray diffraction studies were obtained by slow evaporation of the mother liquid.

The title complex is centrosymmetrical with Mn(II)1 llocated at an inversion centre (Fig. 1, Table 1). Mn(II) was partially oxidized into Mn(III). The three Mn atoms are coordinated octahedrall by four O atoms in the equatorial plane and two pyridine N atoms in axial positions. Mn(II)1 is coordinated by two water molecules and two carboxyl O atoms where ligands act as monodentate bridnging towards Mn(III). The octahedral coordination is completed by two pyridne ligands. Mn(III)2 and Mn(III)2 A reveal the four O atoms from bidentate ligands and two axially positioned pyridine ligands. In the structure, there are intramolecular C—H···π interactions between axially positioned py ligands (Table 2). The complex exhibits a two-dimensional hydrogen bonding network *via* intermolecular interactions O—H..O between coordinated and solvent water molecules, and solvent water and ligand carbonyl group (Table 1, Fig. 2).

### Experimental

MnCl_2 4_H_2_O (0.2 mmol 0.040 g) was dissolved in 5 a ml MeOH and a solution of (0.15 mmol 0.0305 g) of 1-hydroxy-naphthoic acid and (0.30 mmol 0.02 g) MeONa in 10 ml py was added dropwise. The reaction mixture was stirred for 4 h until the solution colour became brown. The mixture was filtered and black-brown single crystals were obtained by slow evaporation of the mother liquid for three weeks at room temperature. m.p.>573 K. Elemental analysis for C_84_H_72_Mn_3_N_8_O_16_ calculated: C 62.50, H 4.50 N 6.94%; found: C 62.43, H 4.23, N 6.81%.

### Refinement

All H atoms were placed geometrically and treated as riding on their parent atoms with C—H 0.93 Å (Phenyl and water) [*U*_iso_(H) = 1.2*U*_eq_(C)].

### Crystal data

**Table:**

[Mn_3_(C_11_H_6_O_3_)_4_(C_5_H_5_N)_6_(H_2_O)_2_]·2H_2_O·2C_5_H_5_N	*Z* = 1
*M**_r_* = 1614.32	*F*_000_ = 835
Triclinic, *P*1	*D*_x_ = 1.372 Mg m^−^^3^
Hall symbol: 1-P	Mo *K*α radiation λ = 0.71073 Å
*a* = 9.962 (3) Å	Cell parameters from 2741 reflections
*b* = 10.170 (3) Å	θ = 2.5–24.1º
*c* = 19.812 (5) Å	µ = 0.55 mm^−^^1^
α = 77.624 (3)º	*T* = 298 (2) K
β = 89.053 (4)º	Block, brown
γ = 85.370 (4)º	0.45 × 0.44 × 0.16 mm
*V* = 1954.2 (10) Å^3^	

### Data collection

**Table:**

Bruker SMART CCD area-detector diffractometer	6766 independent reflections
Radiation source: fine-focus sealed tube	3917 reflections with *I* > 2σ(*I*)
Monochromator: graphite	*R*_int_ = 0.028
*T* = 298(2) K	θ_max_ = 25.0º
φ and ω scans	θ_min_ = 2.1º
Absorption correction: multi-scan(SADABS; Sheldrick, 1996)	*h* = −10→11
*T*_min_ = 0.790, *T*_max_ = 0.917	*k* = −12→11
10152 measured reflections	*l* = −23→23

### Refinement

**Table:**

Refinement on *F*^2^	Secondary atom site location: difference Fourier map
Least-squares matrix: full	Hydrogen site location: inferred from neighbouring sites
*R*[*F*^2^ > 2σ(*F*^2^)] = 0.048	H-atom parameters constrained
*wR*(*F*^2^) = 0.154	*w* = 1/[σ^2^(*F*_o_^2^) + (0.0726*P*)^2^ + 0.4474*P*] where *P* = (*F*_o_^2^ + 2*F*_c_^2^)/3
*S* = 1.00	(Δ/σ)_max_ = 0.001
6766 reflections	Δρ_max_ = 0.41 e Å^−^^3^
502 parameters	Δρ_min_ = −0.26 e Å^−^^3^
744 restraints	Extinction correction: none
Primary atom site location: structure-invariant direct methods	

### Special details

**Table:**

Geometry. All e.s.d.'s (except the e.s.d. in the dihedral angle between two l.s. planes) are estimated using the full covariance matrix. The cell e.s.d.'s are taken into account individually in the estimation of e.s.d.'s in distances, angles and torsion angles; correlations between e.s.d.'s in cell parameters are only used when they are defined by crystal symmetry. An approximate (isotropic) treatment of cell e.s.d.'s is used for estimating e.s.d.'s involving l.s. planes.
Refinement. Refinement of *F*^2^ against ALL reflections. The weighted *R*-factor *wR* and goodness of fit *S* are based on *F*^2^, conventional *R*-factors *R* are based on *F*, with *F* set to zero for negative *F*^2^. The threshold expression of *F*^2^ > σ(*F*^2^) is used only for calculating *R*-factors(gt) *etc*. and is not relevant to the choice of reflections for refinement. *R*-factors based on *F*^2^ are statistically about twice as large as those based on *F*, and *R*- factors based on ALL data will be even larger.

### Fractional atomic coordinates and isotropic or equivalent isotropic displacement parameters (Å^2^)

**Table:**

	*x*	*y*	*z*	*U*_iso_*/*U*_eq_	
Mn1	0.5000	1.0000	1.0000	0.0415 (2)	
Mn2	0.31927 (6)	0.89477 (5)	0.79249 (3)	0.0442 (2)	
N1	0.6800 (3)	1.1017 (3)	0.94749 (15)	0.0452 (8)	
N2	0.5084 (4)	0.9964 (3)	0.73905 (17)	0.0526 (9)	
N3	0.1291 (4)	0.7993 (3)	0.84414 (19)	0.0586 (10)	
N4	0.3397 (9)	0.2282 (9)	0.4436 (5)	0.181 (3)	
O1	0.3432 (3)	0.9767 (2)	0.86811 (12)	0.0504 (7)	
O2	0.3692 (3)	1.1346 (2)	0.92360 (13)	0.0473 (7)	
O3	0.2133 (3)	1.0462 (2)	0.74539 (12)	0.0461 (7)	
O4	0.4274 (3)	0.7444 (2)	0.84399 (12)	0.0516 (7)	
O5	0.5211 (3)	0.5389 (3)	0.87570 (13)	0.0647 (9)	
O6	0.3025 (3)	0.8103 (2)	0.71921 (12)	0.0485 (7)	
O7	0.4426 (3)	1.1701 (3)	1.04953 (13)	0.0540 (8)	
H7A	0.4253	1.2434	1.0201	0.065*	
H7B	0.5034	1.1806	1.0771	0.065*	
O8	0.6500 (3)	0.5604 (3)	0.00202 (16)	0.0808 (10)	
H8A	0.6107	0.5533	−0.0346	0.097*	
H8B	0.5999	0.5317	0.0363	0.097*	
C1	0.3224 (4)	1.1007 (3)	0.87274 (18)	0.0366 (9)	
C2	0.1979 (4)	1.1666 (3)	0.76049 (18)	0.0358 (8)	
C3	0.2463 (4)	1.1986 (3)	0.81975 (17)	0.0358 (8)	
C4	0.2202 (4)	1.3317 (3)	0.83051 (19)	0.0431 (9)	
H4	0.2528	1.3525	0.8704	0.052*	
C5	0.1499 (4)	1.4291 (4)	0.7850 (2)	0.0525 (11)	
H5	0.1335	1.5151	0.7939	0.063*	
C6	0.1009 (4)	1.4007 (4)	0.7235 (2)	0.0453 (10)	
C7	0.1253 (4)	1.2701 (3)	0.71042 (18)	0.0402 (9)	
C8	0.0810 (4)	1.2435 (4)	0.6485 (2)	0.0518 (10)	
H8	0.0955	1.1569	0.6402	0.062*	
C9	0.0164 (5)	1.3439 (4)	0.6000 (2)	0.0657 (13)	
H9	−0.0096	1.3263	0.5581	0.079*	
C10	−0.0104 (5)	1.4724 (4)	0.6134 (3)	0.0733 (14)	
H10	−0.0569	1.5395	0.5809	0.088*	
C11	0.0302 (4)	1.5004 (4)	0.6728 (2)	0.0606 (12)	
H11	0.0115	1.5869	0.6808	0.073*	
C12	0.4649 (4)	0.6277 (4)	0.83032 (19)	0.0453 (10)	
C13	0.3609 (4)	0.6937 (3)	0.71167 (18)	0.0381 (9)	
C14	0.4379 (4)	0.6037 (3)	0.76172 (18)	0.0411 (9)	
C15	0.4924 (5)	0.4784 (4)	0.7471 (2)	0.0562 (11)	
H15	0.5441	0.4180	0.7804	0.067*	
C16	0.4701 (5)	0.4461 (4)	0.6858 (2)	0.0632 (12)	
H16	0.5054	0.3631	0.6782	0.076*	
C17	0.3950 (4)	0.5346 (4)	0.6335 (2)	0.0511 (10)	
C18	0.3395 (4)	0.6602 (4)	0.64584 (18)	0.0418 (9)	
C19	0.2650 (4)	0.7498 (4)	0.59377 (19)	0.0510 (10)	
H19	0.2290	0.8323	0.6018	0.061*	
C20	0.2445 (5)	0.7181 (5)	0.5313 (2)	0.0625 (12)	
H20	0.1954	0.7787	0.4970	0.075*	
C21	0.2978 (5)	0.5939 (5)	0.5194 (2)	0.0700 (13)	
H21	0.2831	0.5719	0.4771	0.084*	
C22	0.3703 (5)	0.5054 (5)	0.5685 (2)	0.0666 (12)	
H22	0.4049	0.4234	0.5593	0.080*	
C23	0.7989 (5)	1.0328 (4)	0.9457 (2)	0.0593 (12)	
H23	0.8072	0.9416	0.9668	0.071*	
C24	0.9085 (5)	1.0897 (5)	0.9146 (3)	0.0766 (14)	
H24	0.9901	1.0383	0.9147	0.092*	
C25	0.8976 (5)	1.2238 (5)	0.8829 (3)	0.0782 (15)	
H25	0.9711	1.2649	0.8607	0.094*	
C26	0.7758 (5)	1.2958 (5)	0.8849 (3)	0.0727 (14)	
H26	0.7652	1.3873	0.8646	0.087*	
C27	0.6708 (5)	1.2312 (4)	0.9171 (2)	0.0558 (11)	
H27	0.5882	1.2805	0.9179	0.067*	
C28	0.5048 (5)	1.0388 (5)	0.6709 (2)	0.0659 (12)	
H28	0.4306	1.0211	0.6474	0.079*	
C29	0.6030 (6)	1.1066 (5)	0.6333 (3)	0.0772 (14)	
H29	0.5966	1.1331	0.5855	0.093*	
C30	0.7098 (6)	1.1345 (5)	0.6673 (3)	0.0806 (15)	
H30	0.7779	1.1822	0.6433	0.097*	
C31	0.7167 (6)	1.0922 (6)	0.7368 (3)	0.0860 (16)	
H31	0.7901	1.1091	0.7611	0.103*	
C32	0.6142 (6)	1.0244 (5)	0.7706 (3)	0.0743 (14)	
H32	0.6194	0.9966	0.8184	0.089*	
C33	0.0188 (6)	0.8033 (5)	0.8077 (3)	0.0890 (16)	
H33	0.0184	0.8478	0.7615	0.107*	
C34	−0.0945 (7)	0.7457 (7)	0.8342 (4)	0.116 (2)	
H34	−0.1699	0.7496	0.8066	0.139*	
C35	−0.0954 (8)	0.6816 (7)	0.9025 (5)	0.121 (2)	
H35	−0.1709	0.6406	0.9223	0.145*	
C36	0.0160 (9)	0.6797 (7)	0.9401 (4)	0.116 (2)	
H36	0.0184	0.6380	0.9868	0.139*	
C37	0.1250 (6)	0.7392 (5)	0.9095 (3)	0.0863 (16)	
H37	0.2011	0.7371	0.9364	0.104*	
C38	0.2680 (11)	0.1573 (9)	0.4938 (5)	0.148 (3)	
H38	0.2957	0.1435	0.5395	0.178*	
C39	0.1524 (9)	0.1041 (8)	0.4777 (5)	0.129 (3)	
H39	0.1008	0.0546	0.5121	0.154*	
C40	0.1193 (8)	0.1246 (8)	0.4157 (6)	0.122 (2)	
H40	0.0417	0.0876	0.4054	0.147*	
C41	0.1857 (10)	0.1948 (8)	0.3629 (4)	0.123 (2)	
H41	0.1551	0.2068	0.3178	0.148*	
C42	0.2955 (9)	0.2465 (8)	0.3768 (5)	0.124 (2)	
H42	0.3440	0.2960	0.3410	0.148*	

### Atomic displacement parameters (Å^2^)

**Table:**

	*U*^11^	*U*^22^	*U*^33^	*U*^12^	*U*^13^	*U*^23^
Mn1	0.0437 (6)	0.0356 (4)	0.0429 (5)	0.0080 (4)	−0.0034 (4)	−0.0073 (4)
Mn2	0.0604 (5)	0.0328 (3)	0.0378 (3)	0.0130 (3)	−0.0127 (3)	−0.0092 (2)
N1	0.042 (2)	0.0400 (18)	0.0497 (19)	0.0071 (16)	−0.0019 (16)	−0.0046 (15)
N2	0.057 (2)	0.054 (2)	0.046 (2)	0.0013 (18)	−0.0064 (18)	−0.0109 (16)
N3	0.067 (3)	0.048 (2)	0.058 (2)	0.0035 (19)	0.005 (2)	−0.0100 (18)
N4	0.188 (8)	0.189 (8)	0.167 (8)	0.002 (6)	0.045 (7)	−0.046 (6)
O1	0.075 (2)	0.0348 (14)	0.0398 (15)	0.0160 (14)	−0.0166 (14)	−0.0110 (11)
O2	0.0583 (19)	0.0387 (14)	0.0457 (16)	0.0068 (13)	−0.0129 (14)	−0.0137 (12)
O3	0.0611 (19)	0.0338 (14)	0.0426 (15)	0.0101 (13)	−0.0202 (13)	−0.0097 (11)
O4	0.073 (2)	0.0371 (14)	0.0428 (15)	0.0197 (14)	−0.0171 (14)	−0.0129 (12)
O5	0.100 (3)	0.0412 (15)	0.0468 (17)	0.0294 (16)	−0.0212 (16)	−0.0079 (13)
O6	0.066 (2)	0.0371 (14)	0.0419 (15)	0.0150 (13)	−0.0146 (13)	−0.0122 (11)
O7	0.065 (2)	0.0497 (16)	0.0483 (16)	0.0146 (14)	−0.0200 (14)	−0.0168 (13)
O8	0.089 (3)	0.073 (2)	0.077 (2)	−0.010 (2)	−0.0011 (19)	−0.0094 (17)
C1	0.039 (2)	0.0328 (19)	0.038 (2)	0.0040 (17)	−0.0018 (17)	−0.0089 (16)
C2	0.036 (2)	0.0284 (18)	0.0398 (19)	0.0022 (16)	−0.0024 (16)	−0.0024 (15)
C3	0.034 (2)	0.0323 (18)	0.0386 (19)	0.0007 (16)	−0.0040 (16)	−0.0039 (15)
C4	0.047 (2)	0.035 (2)	0.048 (2)	0.0024 (18)	−0.0070 (18)	−0.0108 (17)
C5	0.057 (3)	0.0304 (19)	0.068 (3)	0.0041 (19)	−0.005 (2)	−0.0097 (18)
C6	0.042 (2)	0.0334 (19)	0.054 (2)	0.0041 (17)	−0.0057 (19)	0.0019 (17)
C7	0.036 (2)	0.0355 (19)	0.045 (2)	0.0013 (17)	−0.0079 (17)	0.0000 (16)
C8	0.055 (3)	0.045 (2)	0.052 (2)	0.001 (2)	−0.012 (2)	−0.0017 (18)
C9	0.069 (3)	0.060 (3)	0.061 (3)	0.003 (2)	−0.021 (2)	0.003 (2)
C10	0.074 (3)	0.053 (3)	0.078 (3)	0.007 (2)	−0.027 (3)	0.016 (2)
C11	0.059 (3)	0.041 (2)	0.074 (3)	0.011 (2)	−0.013 (2)	0.002 (2)
C12	0.059 (3)	0.033 (2)	0.042 (2)	0.0077 (19)	−0.002 (2)	−0.0076 (17)
C13	0.043 (2)	0.0323 (19)	0.039 (2)	0.0007 (17)	0.0023 (17)	−0.0086 (15)
C14	0.047 (2)	0.0343 (19)	0.040 (2)	0.0048 (18)	−0.0011 (18)	−0.0078 (16)
C15	0.068 (3)	0.045 (2)	0.055 (2)	0.017 (2)	−0.006 (2)	−0.0174 (19)
C16	0.078 (3)	0.047 (2)	0.067 (3)	0.016 (2)	−0.004 (2)	−0.026 (2)
C17	0.053 (3)	0.053 (2)	0.050 (2)	0.002 (2)	0.005 (2)	−0.0205 (19)
C18	0.042 (2)	0.044 (2)	0.041 (2)	−0.0040 (18)	0.0031 (18)	−0.0136 (17)
C19	0.058 (3)	0.049 (2)	0.047 (2)	−0.001 (2)	−0.003 (2)	−0.0132 (18)
C20	0.072 (3)	0.069 (3)	0.049 (2)	−0.003 (2)	−0.008 (2)	−0.017 (2)
C21	0.076 (3)	0.084 (3)	0.057 (3)	−0.002 (3)	−0.004 (2)	−0.033 (2)
C22	0.076 (3)	0.068 (3)	0.063 (3)	0.006 (2)	0.003 (2)	−0.035 (2)
C23	0.048 (3)	0.051 (2)	0.075 (3)	0.007 (2)	−0.001 (2)	−0.008 (2)
C24	0.047 (3)	0.082 (3)	0.095 (4)	0.011 (3)	0.005 (3)	−0.013 (3)
C25	0.054 (3)	0.079 (3)	0.095 (4)	−0.014 (3)	0.012 (3)	−0.002 (3)
C26	0.063 (3)	0.058 (3)	0.089 (3)	−0.004 (3)	0.006 (3)	0.002 (3)
C27	0.052 (3)	0.047 (2)	0.065 (3)	0.005 (2)	0.003 (2)	−0.007 (2)
C28	0.074 (3)	0.067 (3)	0.055 (3)	−0.003 (3)	−0.005 (2)	−0.008 (2)
C29	0.084 (4)	0.078 (3)	0.065 (3)	−0.002 (3)	0.006 (3)	−0.007 (3)
C30	0.073 (4)	0.073 (3)	0.092 (4)	−0.007 (3)	0.018 (3)	−0.011 (3)
C31	0.065 (4)	0.106 (4)	0.092 (4)	−0.015 (3)	−0.006 (3)	−0.029 (3)
C32	0.072 (4)	0.087 (3)	0.064 (3)	−0.002 (3)	−0.007 (3)	−0.016 (3)
C33	0.074 (4)	0.078 (4)	0.111 (4)	−0.001 (3)	0.003 (4)	−0.013 (3)
C34	0.078 (4)	0.103 (5)	0.168 (6)	−0.004 (4)	0.007 (5)	−0.033 (4)
C35	0.103 (5)	0.088 (4)	0.171 (6)	−0.018 (4)	0.064 (5)	−0.029 (4)
C36	0.131 (6)	0.088 (4)	0.120 (5)	−0.016 (4)	0.044 (5)	−0.003 (4)
C37	0.101 (4)	0.073 (3)	0.081 (4)	−0.007 (3)	0.018 (3)	−0.010 (3)
C38	0.141 (7)	0.176 (7)	0.120 (6)	0.000 (6)	0.013 (6)	−0.020 (5)
C39	0.110 (6)	0.113 (5)	0.152 (6)	−0.007 (4)	0.033 (5)	−0.007 (5)
C40	0.086 (5)	0.113 (5)	0.171 (7)	0.017 (4)	0.006 (5)	−0.046 (5)
C41	0.115 (6)	0.117 (5)	0.133 (6)	0.044 (5)	−0.016 (5)	−0.033 (4)
C42	0.121 (6)	0.109 (5)	0.133 (6)	0.013 (5)	0.028 (5)	−0.017 (4)

### Geometric parameters (Å, °)

**Table:**

Mn1—O2	2.180 (2)	C14—C15	1.430 (5)
Mn1—O2^i^	2.180 (2)	C15—C16	1.350 (5)
Mn1—O7^i^	2.198 (2)	C15—H15	0.9300
Mn1—O7	2.198 (2)	C16—C17	1.400 (5)
Mn1—N1^i^	2.268 (3)	C16—H16	0.9300
Mn1—N1	2.268 (3)	C17—C22	1.413 (5)
Mn2—O6	1.854 (2)	C17—C18	1.418 (5)
Mn2—O3	1.874 (2)	C18—C19	1.400 (5)
Mn2—O1	1.891 (2)	C19—C20	1.367 (5)
Mn2—O4	1.909 (2)	C19—H19	0.9300
Mn2—N3	2.321 (4)	C20—C21	1.395 (6)
Mn2—N2	2.349 (4)	C20—H20	0.9300
N1—C27	1.322 (5)	C21—C22	1.349 (6)
N1—C23	1.331 (5)	C21—H21	0.9300
N2—C32	1.315 (6)	C22—H22	0.9300
N2—C28	1.326 (5)	C23—C24	1.353 (6)
N3—C37	1.309 (6)	C23—H23	0.9300
N3—C33	1.319 (6)	C24—C25	1.370 (6)
N4—C38	1.331 (10)	C24—H24	0.9300
N4—C42	1.373 (10)	C25—C26	1.370 (7)
O1—C1	1.284 (4)	C25—H25	0.9300
O2—C1	1.242 (4)	C26—C27	1.357 (6)
O3—C2	1.318 (4)	C26—H26	0.9300
O4—C12	1.297 (4)	C27—H27	0.9300
O5—C12	1.235 (4)	C28—C29	1.361 (6)
O6—C13	1.316 (4)	C28—H28	0.9300
O7—H7A	0.8500	C29—C30	1.350 (7)
O7—H7B	0.8501	C29—H29	0.9300
O8—H8A	0.8500	C30—C31	1.351 (7)
O8—H8B	0.8500	C30—H30	0.9300
C1—C3	1.458 (5)	C31—C32	1.363 (7)
C2—C3	1.388 (5)	C31—H31	0.9300
C2—C7	1.438 (5)	C32—H32	0.9300
C3—C4	1.418 (5)	C33—C34	1.359 (8)
C4—C5	1.346 (5)	C33—H33	0.9300
C4—H4	0.9300	C34—C35	1.371 (9)
C5—C6	1.413 (5)	C34—H34	0.9300
C5—H5	0.9300	C35—C36	1.343 (9)
C6—C7	1.408 (5)	C35—H35	0.9300
C6—C11	1.416 (5)	C36—C37	1.357 (8)
C7—C8	1.398 (5)	C36—H36	0.9300
C8—C9	1.369 (5)	C37—H37	0.9300
C8—H8	0.9300	C38—C39	1.381 (10)
C9—C10	1.393 (6)	C38—H38	0.9300
C9—H9	0.9300	C39—C40	1.247 (10)
C10—C11	1.346 (6)	C39—H39	0.9300
C10—H10	0.9300	C40—C41	1.332 (10)
C11—H11	0.9300	C40—H40	0.9300
C12—C14	1.464 (5)	C41—C42	1.308 (10)
C13—C14	1.390 (5)	C41—H41	0.9300
C13—C18	1.440 (5)	C42—H42	0.9300
			
O2—Mn1—O2^i^	180.00 (11)	C13—C14—C15	119.1 (3)
O2—Mn1—O7^i^	103.30 (9)	C13—C14—C12	123.9 (3)
O2^i^—Mn1—O7^i^	76.70 (9)	C15—C14—C12	117.0 (3)
O2—Mn1—O7	76.70 (9)	C16—C15—C14	121.2 (4)
O2^i^—Mn1—O7	103.30 (9)	C16—C15—H15	119.4
O7^i^—Mn1—O7	180.00 (10)	C14—C15—H15	119.4
O2—Mn1—N1^i^	90.93 (11)	C15—C16—C17	121.6 (4)
O2^i^—Mn1—N1^i^	89.07 (11)	C15—C16—H16	119.2
O7^i^—Mn1—N1^i^	92.12 (11)	C17—C16—H16	119.2
O7—Mn1—N1^i^	87.88 (11)	C16—C17—C22	123.4 (4)
O2—Mn1—N1	89.07 (11)	C16—C17—C18	119.0 (4)
O2^i^—Mn1—N1	90.93 (11)	C22—C17—C18	117.5 (4)
O7^i^—Mn1—N1	87.88 (11)	C19—C18—C17	119.5 (3)
O7—Mn1—N1	92.12 (11)	C19—C18—C13	121.0 (3)
N1^i^—Mn1—N1	180.0	C17—C18—C13	119.5 (3)
O6—Mn2—O3	90.31 (10)	C20—C19—C18	121.0 (4)
O6—Mn2—O1	177.65 (12)	C20—C19—H19	119.5
O3—Mn2—O1	91.78 (10)	C18—C19—H19	119.5
O6—Mn2—O4	92.30 (10)	C19—C20—C21	119.4 (4)
O3—Mn2—O4	177.38 (10)	C19—C20—H20	120.3
O1—Mn2—O4	85.61 (10)	C21—C20—H20	120.3
O6—Mn2—N3	90.47 (13)	C22—C21—C20	121.0 (4)
O3—Mn2—N3	90.79 (13)	C22—C21—H21	119.5
O1—Mn2—N3	90.58 (13)	C20—C21—H21	119.5
O4—Mn2—N3	89.02 (13)	C21—C22—C17	121.5 (4)
O6—Mn2—N2	89.72 (12)	C21—C22—H22	119.3
O3—Mn2—N2	87.78 (12)	C17—C22—H22	119.3
O1—Mn2—N2	89.28 (12)	N1—C23—C24	122.9 (4)
O4—Mn2—N2	92.40 (12)	N1—C23—H23	118.6
N3—Mn2—N2	178.56 (12)	C24—C23—H23	118.6
C27—N1—C23	117.4 (4)	C23—C24—C25	119.2 (5)
C27—N1—Mn1	121.4 (3)	C23—C24—H24	120.4
C23—N1—Mn1	121.1 (3)	C25—C24—H24	120.4
C32—N2—C28	116.2 (4)	C26—C25—C24	118.3 (5)
C32—N2—Mn2	126.2 (3)	C26—C25—H25	120.8
C28—N2—Mn2	117.3 (3)	C24—C25—H25	120.8
C37—N3—C33	116.8 (5)	C27—C26—C25	118.8 (4)
C37—N3—Mn2	122.8 (4)	C27—C26—H26	120.6
C33—N3—Mn2	120.4 (4)	C25—C26—H26	120.6
C38—N4—C42	118.3 (10)	N1—C27—C26	123.3 (4)
C1—O1—Mn2	130.2 (2)	N1—C27—H27	118.3
C1—O2—Mn1	123.5 (2)	C26—C27—H27	118.3
C2—O3—Mn2	127.3 (2)	N2—C28—C29	124.0 (5)
C12—O4—Mn2	130.9 (2)	N2—C28—H28	118.0
C13—O6—Mn2	127.9 (2)	C29—C28—H28	118.0
Mn1—O7—H7A	112.0	C30—C29—C28	118.2 (5)
Mn1—O7—H7B	111.5	C30—C29—H29	120.9
H7A—O7—H7B	109.6	C28—C29—H29	120.9
H8A—O8—H8B	108.4	C29—C30—C31	119.2 (5)
O2—C1—O1	118.0 (3)	C29—C30—H30	120.4
O2—C1—C3	121.1 (3)	C31—C30—H30	120.4
O1—C1—C3	120.9 (3)	C30—C31—C32	119.0 (5)
O3—C2—C3	124.9 (3)	C30—C31—H31	120.5
O3—C2—C7	115.8 (3)	C32—C31—H31	120.5
C3—C2—C7	119.2 (3)	N2—C32—C31	123.4 (5)
C2—C3—C4	119.3 (3)	N2—C32—H32	118.3
C2—C3—C1	123.0 (3)	C31—C32—H32	118.3
C4—C3—C1	117.7 (3)	N3—C33—C34	123.3 (6)
C5—C4—C3	122.2 (3)	N3—C33—H33	118.3
C5—C4—H4	118.9	C34—C33—H33	118.3
C3—C4—H4	118.9	C33—C34—C35	118.7 (7)
C4—C5—C6	120.0 (3)	C33—C34—H34	120.6
C4—C5—H5	120.0	C35—C34—H34	120.6
C6—C5—H5	120.0	C36—C35—C34	118.1 (7)
C7—C6—C5	119.8 (3)	C36—C35—H35	121.0
C7—C6—C11	117.9 (4)	C34—C35—H35	121.0
C5—C6—C11	122.3 (4)	C35—C36—C37	119.4 (7)
C8—C7—C6	119.6 (3)	C35—C36—H36	120.3
C8—C7—C2	120.9 (3)	C37—C36—H36	120.3
C6—C7—C2	119.4 (3)	N3—C37—C36	123.7 (6)
C9—C8—C7	120.5 (4)	N3—C37—H37	118.2
C9—C8—H8	119.7	C36—C37—H37	118.2
C7—C8—H8	119.7	N4—C38—C39	119.8 (9)
C8—C9—C10	119.9 (4)	N4—C38—H38	120.1
C8—C9—H9	120.0	C39—C38—H38	120.1
C10—C9—H9	120.0	C40—C39—C38	117.9 (9)
C11—C10—C9	120.7 (4)	C40—C39—H39	121.0
C11—C10—H10	119.7	C38—C39—H39	121.0
C9—C10—H10	119.7	C39—C40—C41	125.5 (10)
C10—C11—C6	121.2 (4)	C39—C40—H40	117.2
C10—C11—H11	119.4	C41—C40—H40	117.2
C6—C11—H11	119.4	C42—C41—C40	117.7 (9)
O5—C12—O4	119.3 (3)	C42—C41—H41	121.1
O5—C12—C14	121.5 (3)	C40—C41—H41	121.1
O4—C12—C14	119.2 (3)	C41—C42—N4	120.6 (9)
O6—C13—C14	125.1 (3)	C41—C42—H42	119.7
O6—C13—C18	115.3 (3)	N4—C42—H42	119.7
C14—C13—C18	119.6 (3)		
			
O2—Mn1—N1—C27	32.6 (3)	C5—C6—C7—C2	1.1 (6)
O2^i^—Mn1—N1—C27	−147.4 (3)	C11—C6—C7—C2	179.5 (4)
O7^i^—Mn1—N1—C27	136.0 (3)	O3—C2—C7—C8	−3.0 (5)
O7—Mn1—N1—C27	−44.0 (3)	C3—C2—C7—C8	176.7 (4)
N1^i^—Mn1—N1—C27	153 (100)	O3—C2—C7—C6	178.2 (3)
O2—Mn1—N1—C23	−147.0 (3)	C3—C2—C7—C6	−2.1 (6)
O2^i^—Mn1—N1—C23	33.0 (3)	C6—C7—C8—C9	1.2 (6)
O7^i^—Mn1—N1—C23	−43.6 (3)	C2—C7—C8—C9	−177.6 (4)
O7—Mn1—N1—C23	136.4 (3)	C7—C8—C9—C10	−2.6 (7)
N1^i^—Mn1—N1—C23	−27 (100)	C8—C9—C10—C11	2.0 (8)
O6—Mn2—N2—C32	−146.6 (4)	C9—C10—C11—C6	−0.1 (8)
O3—Mn2—N2—C32	123.1 (4)	C7—C6—C11—C10	−1.2 (7)
O1—Mn2—N2—C32	31.3 (4)	C5—C6—C11—C10	177.1 (4)
O4—Mn2—N2—C32	−54.3 (4)	Mn2—O4—C12—O5	−170.7 (3)
N3—Mn2—N2—C32	116 (5)	Mn2—O4—C12—C14	8.4 (6)
O6—Mn2—N2—C28	38.7 (3)	Mn2—O6—C13—C14	6.7 (6)
O3—Mn2—N2—C28	−51.6 (3)	Mn2—O6—C13—C18	−173.9 (3)
O1—Mn2—N2—C28	−143.4 (3)	O6—C13—C14—C15	178.2 (4)
O4—Mn2—N2—C28	131.0 (3)	C18—C13—C14—C15	−1.1 (6)
N3—Mn2—N2—C28	−59 (5)	O6—C13—C14—C12	−0.2 (6)
O6—Mn2—N3—C37	132.4 (4)	C18—C13—C14—C12	−179.6 (4)
O3—Mn2—N3—C37	−137.3 (4)	O5—C12—C14—C13	171.8 (4)
O1—Mn2—N3—C37	−45.5 (4)	O4—C12—C14—C13	−7.2 (6)
O4—Mn2—N3—C37	40.1 (4)	O5—C12—C14—C15	−6.7 (6)
N2—Mn2—N3—C37	−130 (5)	O4—C12—C14—C15	174.2 (4)
O6—Mn2—N3—C33	−47.2 (4)	C13—C14—C15—C16	−0.1 (7)
O3—Mn2—N3—C33	43.1 (4)	C12—C14—C15—C16	178.5 (4)
O1—Mn2—N3—C33	134.9 (4)	C14—C15—C16—C17	1.2 (7)
O4—Mn2—N3—C33	−139.5 (4)	C15—C16—C17—C22	179.4 (5)
N2—Mn2—N3—C33	50 (5)	C15—C16—C17—C18	−1.0 (7)
O6—Mn2—O1—C1	137 (3)	C16—C17—C18—C19	179.6 (4)
O3—Mn2—O1—C1	−15.6 (4)	C22—C17—C18—C19	−0.8 (6)
O4—Mn2—O1—C1	164.6 (4)	C16—C17—C18—C13	−0.3 (6)
N3—Mn2—O1—C1	−106.4 (3)	C22—C17—C18—C13	179.4 (4)
N2—Mn2—O1—C1	72.1 (3)	O6—C13—C18—C19	2.1 (5)
O2^i^—Mn1—O2—C1	−121 (100)	C14—C13—C18—C19	−178.5 (4)
O7^i^—Mn1—O2—C1	12.9 (3)	O6—C13—C18—C17	−178.1 (3)
O7—Mn1—O2—C1	−167.1 (3)	C14—C13—C18—C17	1.3 (6)
N1^i^—Mn1—O2—C1	−79.5 (3)	C17—C18—C19—C20	0.3 (6)
N1—Mn1—O2—C1	100.5 (3)	C13—C18—C19—C20	−179.9 (4)
O6—Mn2—O3—C2	−166.6 (3)	C18—C19—C20—C21	0.5 (7)
O1—Mn2—O3—C2	12.3 (3)	C19—C20—C21—C22	−0.6 (7)
O4—Mn2—O3—C2	17 (3)	C20—C21—C22—C17	0.1 (8)
N3—Mn2—O3—C2	102.9 (3)	C16—C17—C22—C21	−179.7 (5)
N2—Mn2—O3—C2	−76.9 (3)	C18—C17—C22—C21	0.6 (7)
O6—Mn2—O4—C12	−2.8 (4)	C27—N1—C23—C24	0.0 (7)
O3—Mn2—O4—C12	173 (3)	Mn1—N1—C23—C24	179.6 (4)
O1—Mn2—O4—C12	178.3 (4)	N1—C23—C24—C25	−0.4 (8)
N3—Mn2—O4—C12	87.6 (4)	C23—C24—C25—C26	0.9 (8)
N2—Mn2—O4—C12	−92.6 (4)	C24—C25—C26—C27	−1.0 (8)
O3—Mn2—O6—C13	175.2 (3)	C23—N1—C27—C26	−0.1 (6)
O1—Mn2—O6—C13	23 (3)	Mn1—N1—C27—C26	−179.7 (3)
O4—Mn2—O6—C13	−4.9 (3)	C25—C26—C27—N1	0.6 (7)
N3—Mn2—O6—C13	−94.0 (3)	C32—N2—C28—C29	0.6 (7)
N2—Mn2—O6—C13	87.5 (3)	Mn2—N2—C28—C29	175.9 (4)
Mn1—O2—C1—O1	6.0 (5)	N2—C28—C29—C30	−1.1 (8)
Mn1—O2—C1—C3	−174.0 (2)	C28—C29—C30—C31	1.2 (8)
Mn2—O1—C1—O2	−166.6 (3)	C29—C30—C31—C32	−1.0 (8)
Mn2—O1—C1—C3	13.3 (5)	C28—N2—C32—C31	−0.4 (7)
Mn2—O3—C2—C3	−7.9 (5)	Mn2—N2—C32—C31	−175.2 (4)
Mn2—O3—C2—C7	171.8 (2)	C30—C31—C32—N2	0.7 (8)
O3—C2—C3—C4	−178.7 (4)	C37—N3—C33—C34	−1.8 (8)
C7—C2—C3—C4	1.7 (5)	Mn2—N3—C33—C34	177.8 (4)
O3—C2—C3—C1	0.5 (6)	N3—C33—C34—C35	0.9 (10)
C7—C2—C3—C1	−179.1 (3)	C33—C34—C35—C36	0.4 (10)
O2—C1—C3—C2	176.8 (4)	C34—C35—C36—C37	−0.7 (11)
O1—C1—C3—C2	−3.1 (6)	C33—N3—C37—C36	1.5 (8)
O2—C1—C3—C4	−4.0 (5)	Mn2—N3—C37—C36	−178.2 (4)
O1—C1—C3—C4	176.1 (4)	C35—C36—C37—N3	−0.2 (10)
C2—C3—C4—C5	−0.2 (6)	C42—N4—C38—C39	0.1 (13)
C1—C3—C4—C5	−179.4 (4)	N4—C38—C39—C40	0.3 (14)
C3—C4—C5—C6	−0.9 (6)	C38—C39—C40—C41	−0.6 (13)
C4—C5—C6—C7	0.4 (6)	C39—C40—C41—C42	0.4 (13)
C4—C5—C6—C11	−177.9 (4)	C40—C41—C42—N4	0.0 (12)
C5—C6—C7—C8	−177.7 (4)	C38—N4—C42—C41	−0.2 (13)
C11—C6—C7—C8	0.6 (6)		

Symmetry codes: (i) −*x*+1, −*y*+2, −*z*+2.

### Hydrogen-bond geometry (Å, °)

**Table:**

*D*—H···*A*	*D*—H	H···*A*	*D*···*A*	*D*—H···*A*
O7—H7A···O8^ii^	0.85	2.03	2.799	150
O8—H8A···O5^iii^	0.85	2.04	2.889	178
O8—H8B···O5^iv^	0.85	2.13	2.981	178
C31—H31···Cg^v^	0.93	3.22	3.847	127

Symmetry codes: (ii) −*x*+1, −*y*+2, −*z*+1; (iii) *x*, *y*, *z*−1; (iv) −*x*+1, −*y*+1, −*z*+1; (v) −*x*+2, −*y*+2, −*z*+2.
